# SNP interaction detection with Random Forests in high-dimensional genetic data

**DOI:** 10.1186/1471-2105-13-164

**Published:** 2012-07-15

**Authors:** Stacey J Winham, Colin L Colby, Robert R Freimuth, Xin Wang, Mariza de Andrade, Marianne Huebner, Joanna M Biernacka

**Affiliations:** 1 Department of Health Sciences Research, Mayo Clinic, 200 First Street Southwest, Rochester, MN, 55905, USA; 2 Department of Statistics and Probability, Michigan State University, A413 Wells Hall, East Lansing, MI, 48824, USA; 3 Department of Psychiatry and Psychology, Mayo Clinic, 200 First Street Southwest, Rochester, MN, 55905, USA

## Abstract

**Background:**

Identifying variants associated with complex human traits in high-dimensional data is a central goal of genome-wide association studies. However, complicated etiologies such as gene-gene interactions are ignored by the univariate analysis usually applied in these studies. Random Forests (RF) are a popular data-mining technique that can accommodate a large number of predictor variables and allow for complex models with interactions. RF analysis produces measures of variable importance that can be used to rank the predictor variables. Thus, single nucleotide polymorphism (SNP) analysis using RFs is gaining popularity as a potential filter approach that considers interactions in high-dimensional data. However, the impact of data dimensionality on the power of RF to identify interactions has not been thoroughly explored. We investigate the ability of rankings from variable importance measures to detect gene-gene interaction effects and their potential effectiveness as filters compared to p-values from univariate logistic regression, particularly as the data becomes increasingly high-dimensional.

**Results:**

RF effectively identifies interactions in low dimensional data. As the total number of predictor variables increases, probability of detection declines more rapidly for interacting SNPs than for non-interacting SNPs, indicating that in high-dimensional data the RF variable importance measures are capturing marginal effects rather than capturing the effects of interactions.

**Conclusions:**

While RF remains a promising data-mining technique that extends univariate methods to condition on multiple variables simultaneously, RF variable importance measures fail to detect interaction effects in high-dimensional data in the absence of a strong marginal component, and therefore may not be useful as a filter technique that allows for interaction effects in genome-wide data.

## Background

Genome-wide association studies (GWAS) have been successful in detecting single locus variants with relatively large effects in some common, complex diseases [1,2]. However, risk SNPs identified thus far can explain only a small percentage of the estimated heritability of such traits. This may be partly due to the fact that commonly used single SNP analysis strategies employed in GWAS are designed to detect common variants with strong marginal associations, and are not suitable for detecting complex multigenic disease risk factors, which may account for some of the missing heritability [3-5]. Phenotypic variation present in common, complex diseases is thought to involve complex etiologies including complicated interactions between many genetic and environmental factors [6]. In particular, it is believed that gene-gene interaction effects, or conditional dependence between genetic variants affecting the phenotype, contribute to complex traits. Ignoring those interactions in univariate analyses may be limiting the success of GWAS studies for complex diseases [2,7].

As an alternative to traditional statistical methods that fail to appropriately account for these complex genetic architectures, data-mining approaches designed to discover patterns in large amounts of data are gaining popularity for genetic association studies. Many data-mining and machine learning approaches were developed with a main goal of prediction, such as exhaustive search strategies [8], penalized regression [9], multifactor dimensionality reduction [10], neural networks [11], and support vector machines [12], but many of these methods have also been applied for variable selection or identification of relevant predictor variables (filtering/screening). One commonly-used data-mining technique is Random Forests (RF), which builds an ensemble of classification and regression trees that can predict an outcome (e.g. disease status) based on a large number of predictors (e.g. SNPs), allowing for potentially complex models that can include interactions of the predictor variables [13]. RFs have been proposed for analysis of genetic data [14], and more recently have been suggested as a way of analyzing even large SNP data sets from GWAS [15]. Although designed for prediction, RFs give variable importance measures (VIM) that can be used to rank SNPs, and are therefore gaining popularity as a potential filter approach which considers interactions. In high-dimensional data, RF VIM rankings can be used for screening or filtering by selecting top-ranking SNPs for follow-up study [16]; in this context, RF rankings may have an advantage over univariate approaches because they may better reflect complex disease models.

The performance of RFs in the context of genetic data analysis has been investigated, and RF VIMs have been shown to out-perform Fisher’s exact test as a screening tool when interactions are present [17]. RFs have been utilized in a number of genetic studies, and have recently been applied to a genome-wide association study of multiple sclerosis using filtering and backward elimination techniques, although identification of interactions was not explicitly considered [15]. To facilitate genome-wide application, RFs were iteratively applied for ‘sparsity pruning’ using VIMs to filter out non-predictive SNPs in a backward elimination technique [18].

In addition to performance as a filtering/screening tool, other properties of VIMs have also been previously investigated. For instance, the bias of these measures has been assessed under linkage disequilibrium [19], and an extension has been developed to consider the joint importance of multiple variables [20]. However, previous research has not considered the impact on interaction detection as data dimension (i.e. number of predictor variables) increases.

A frequently cited benefit of RFs in the analysis of genetic data is that they capture interactions between predictor variables, because the hierarchical decision tree structure can model non-linear associations [17]. The RF methodology is not designed to explicitly test for the presence of interactions or individual risk factor effects with a hypothesis test of significance. Nevertheless, the variable importance measures for individual risk factors are expected to assess a variable’s overall impact on prediction and to reflect both main and interaction effects. Methods for estimating the importance of joint effects including interactions (as opposed to individual predictor variables) have been proposed [20]. However, these are not feasible in the context of large genetic datasets consisting of tens or hundreds of thousands of variables and thus interactions are usually not studied explicitly. Yet even without explicit inclusion of interaction effects in the RFs, the individual SNP VIMs used for screening are often claimed to capture interaction effects [21].

Previous studies of RF performance have primarily applied the approach in lower-dimensional settings, or settings involving interactions with strong marginal components. Although it has been shown that in relatively small datasets RFs can detect interacting risk factors better than univariate tests of association of individual predictors [17], this observation may not extend well to large datasets, with many potential predictors in the analysis. In fact, we might expect that the probability to detect true interactions may decrease as dimension increases, because the probability of the co-occurrence of interacting predictors decreases as the feature space grows, and earlier work suggests that RFs may be better at detecting main effects than interactions (due to the independence assumption used in tree construction) [22].

In this study we explore the ability of RF VIMs to capture interaction effects, particularly as the data becomes increasingly high-dimensional. We hypothesize that when standard RF VIMs are used to identify the best predictors, the ability to detect interacting effects will decline rapidly as the total number of studied predictor variables is increased. Focusing on analysis of binary case/control data, where RF is used for classification, this paper presents a simulation study to investigate the relationship between the number of variables in a RF and the ability of the approach to detect both marginal and interaction effects. We compare the performance of various RF measures of variable importance to p-values from univariate logistic regression under different data-generating models for complex disease, both as the number of predictors becomes large and as the strength of marginal association diminishes.

## Methods

### Random forests

Random Forests is an ensemble or ‘forest’ of many classification and regression tree (CART) classifiers [13]. Each tree is constructed on a bootstrap sample of subjects, with a random subset of the total number of predictors, *p,* eligible for selection at each node in the tree. The final prediction or classification is obtained via bootstrap aggregating (bagging) [23], and is based on a vote over all of the trees in the ensemble. In this study, we focus on classification for binary traits rather than regression for quantitative traits.

In general, a Random Forest of decision tree classifiers is grown as follows:

1. Select a total of *ntree* bootstrap samples of size *N* from the original data for training. On average, about one third of the samples are left out, which are called the ‘out-of-bag’ (OOB) data.

2. For each bootstrap sample, grow an unpruned classification or regression tree (CART) [24].

a. At each node in the tree, randomly select *mtry* variables from the total *p* predictor variables.

b. Choose the best split at each node from among the *mtry* variables by maximizing some measure of node purity (degree to which members of a node belong to one class/category of the outcome variable), such as the Gini index [24].

c. An estimate of prediction error (i.e. the probability of misclassification) is obtained for each tree using the OOB individuals.

3. For a given observation, the final prediction/classification is the majority vote (the predicted class in the majority of trees) over all trees in which that observation was ‘out-of-bag’. The OOB prediction error and prediction accuracy of the RF can be calculated by considering accuracy of the OOB prediction over all subjects.

For more detail on the method, see [13].

### Variable importance measures

In addition to providing prediction, the RF method can be used to calculate a VIM for each predictor. Ranking based on VIMs can then be used as a screening tool to prioritize variables for follow-up study. A number of importance measures have been proposed, including Gini importance and mean decrease in accuracy (MDA).

Let *X* be a particular predictor variable (e.g. SNP). The Gini importance of *X* measures the total decrease in node impurity across all nodes in the forest when *X* was selected for splitting [13]. Although easy to compute, this measure has been shown to be biased by preferentially selecting variables with many categories [25]; hence the more computationally intensive MDA variable importance measure is typically preferred. The unscaled MDA is the reduction in prediction accuracy after the data for the variable of interest are permuted across all samples within each tree; this VIM is often referred to as ‘permutation importance’. If a variable is truly predictive of the response, permuting the variable across samples should disrupt this association and result in lower prediction accuracy; conversely, prediction accuracy should not change significantly after permuting data for unassociated variables. First, the prediction accuracy, *A*_*t*_*,* is estimated from the proportion of correctly classified OOB individuals for each tree *t* in the forest *T*. The values of *X* across samples are then randomly permuted in each tree, and the accuracy *A*_t_^*^ is calculated for the OOB individuals using the permuted data. The raw or unscaled MDA is

(1) $$MDA=\frac{1}{ntree}\sum_{t\in T}A_{t}-A_{t}^{*}\text{.}$$

Variable importance can also be measured through a scaled version of MDA,

(2) MDAscaled=MDAs2ntree

where *s*^*2*^ is the estimated variance of decrease in accuracy across trees [26]. It should be noted that for trees in which the predictor variable *X* does not appear, the decrease in accuracy *A*_*t*_*- A*_t_^*^ is zero by definition. Another version of MDA variable importance attributed to Meng only considers trees in which *X* appears [27].

### Interaction detection

Typically VIMs are used to rank variables, and variables with high ranks are considered as potentially associated with the phenotype. A true causative genetic factor may be considered to be identified or detected by the RF analysis if it ranks highly in terms of variable importance, above null factors that are not associated with the phenotype. In our study, we considered a SNP to be detected if it ranked within the top *k* SNPs, where *k* is the number of SNPs with a simulated causal effect. Other definitions of detection were also investigated with similar results, so results are presented for this definition of detection only.

In RF analysis, the importance of each variable takes into account, or is conditional on, the effects of other variables in the tree. However, RF importance measures do not specify whether an effect is marginal or due to interactions with other SNPs. Because one of the frequently cited advantages of RFs is the ability to model interactions, our primary goal was to investigate RF’s ability to detect both the effects of SNPs that act independently as well as those whose influence on the phenotype is dependent on genotypes at another locus (i.e. SNPs with interaction effects).

In order to investigate the performance of RF VIMs for these different types of effects, we need to quantify both the strength and type of a variable’s effect on the phenotype. Heritability in the broad sense, or the proportion of phenotypic variation that can be explained by genetic variation, is a common measure of the degree of genetic determination of a trait, or the total genetic effect size, and can also be used to estimate the effect of a particular disease locus [28]. Suppose a binary disease phenotype *D* is controlled by two susceptibility loci *A* and *B*, with genotypes *a,b* = 0,1,2 at each locus. The total heritability *H*^*2*^ due to the two loci can be defined as:

(3) $$H_{\mathrm{AB}}^{2}=\frac{1}{P\left(D\right)\left(1-P\left(D\right)\right)}\sum_{a=0}^{2}\sum_{b=0}^{2}P\left(G_{\mathrm{ab}}\right){\left(P\left(D|G_{\mathrm{ab}}\right)-P\left(D\right)\right)}^{2}$$

where *P(D)* is the disease prevalence, *P(G*_*ab*_*)* is the frequency of genotype combination *ab*, and *P(D|G*_*ab*_*)* is the penetrance of the disease [29]. We can define the heritability due to the marginal effect of SNP A as

(4) $$H_{M,A}^{2}=\frac{1}{P\left(D\right)\left(1-P\left(D\right)\right)}\sum_{a=0}^{2}\left(\sum_{b=0}^{2}P\left(G_{\mathrm{ab}}\right)\right)\times {\left(\sum_{b=0}^{2}P\left(D|G_{\mathrm{ab}}\right)P\left(G_{\mathrm{ab}}\right)-P\left(D\right)\right)}^{2}$$

Similarly, we can define *H*^*2*^_*M,B*_ for SNP B. The heritability due to the interaction effect of SNP A and SNP B, the conditional dependence of SNPs A and B on the phenotype, can be defined as the portion of the total heritability not attributable to the marginal effects at either locus: $H_{I,AB}^{2}=H_{\mathrm{AB}}^{2}-H_{M,A}^{2}-H_{M,B}^{2}$. Based on these definitions, SNP A will confer a ‘main effect’ on the phenotype if *H*^*2*^_*M,A*_ > 0 and an ‘interaction effect’ if *H*^*2*^_*I,AB*_ > 0 for some SNP B. These ideas can easily be extended to models with more than two causative loci.

### Simulation study design

In order to investigate the performance of RF VIMs in detecting interactions for a binary disease phenotype, we developed a sequence of three simulation studies. Data sets that included variables with main effects only ($H_{I,AB}^{2}=0$) and variables with interaction effects ($H_{I,AB}^{2}>0$) on the phenotype were simulated and the performance of RFs in detecting the effects of these different types of variables was assessed. Because an RF analysis is dependent on the tuning parameters selected, preliminary studies were conducted to determine optimal settings of *mtry* and *ntree* for this study based on prediction error (and detection probability), current recommendations in the literature, and practical considerations ( Additional file 1). Based on these preliminary results, *mtry* = .1*p* and *ntree* = 5,000 were used for the analyses presented here.

The first two simulation studies were designed to assess the performance of RF VIMs for detecting main and interaction effects, and compare the performance of RF with p-value rankings from univariate logistic regression. In order to focus on evaluating the performance of the methods in relation to the strength and type (main vs. interaction) of effect, in these simulations all SNPs were assumed to be independent and had the same minor allele frequencies. The third simulation study investigated the impact of linkage disequilibrium (LD) on the detection of main and interaction effects using RFs. The designs of all three simulations are summarized in Table 1.

**Table 1:** Summary of the objectives and design of simulations 1-3

	**Simulation 1**	**Simulation 2**	**Simulation 3**
**Objective**	To compare RF VIMs for main and interaction effect detection.	To compare RF measures with p-values from logistic regression for main and interaction effect detection.	Examine RF performance in presence of realistic patterns of LD and MAF.
**Independent SNPs**	Yes	Yes	No (LD)
**# Total Loci (*p*)**	10, 100, 500, 1000	10, 100, 500, 1000	Fixed at 1000
**# Causal Loci (*k*)**	4	2	2
**MAF**	Fixed at 0.1, 0.2, 0.3, or 0.4	Fixed at 0.3	Varies (0.01–0.50)
**# Model Scenarios**	5	3	4
**Description**	Varying effect sizes, H_X1X2_^2^ vs. H_X3X4_^2^	Two interacting SNPs with 0, 1, or 2 having main effects.	Causal SNPs chosen in blocks of strong vs. weak LD with non-causal SNPs.
**Phenotype Generation**	Phenotype is a dichotomized quantitative (normally distributed) trait.	Phenotype is based on direct penetrance functions.	Phenotypes are generated as in Simulation 1.

In all simulations, data were generated assuming that some SNPs contribute to the overall heritability only marginally (‘main’), some SNPs contribute both marginally and interactively (‘interacting’), and that some SNPs are not causally associated with the outcome and thus do not contribute to the total heritability (‘null’). The performance of RFs was evaluated for each of the three types of variables by estimating probability of detection, which is similar to the concept of power in a frequentist statistical framework. A SNP effect was considered to be detected if its rank based on the VIM was in the top *k* ranks, where *k* is the total number of causal variants. The probability of detection was compared for a range of simulation scenarios for the three types of variables. Analyses were performed using the software package Random Jungle [26] and results were summarized and plotted using R statistical software.

### Simulation 1: comparing performance of VIMs for detecting main vs. interaction effects

The goal of the first simulation study was to compare the performance of RF VIMs for detecting marginal and interaction effects. The performance of RF was also compared to the most common GWAS analysis approach, univariate logistic regression. The comparisons were performed for independent SNP data with fixed MAF, but with varying degrees of effect size and patterns of interaction. Case/control datasets with *p* total SNPs were generated (*p* = 10, 100, 500, 1000), where *k* = 4 causal SNPs were associated with the binary disease phenotype, *D*, with two SNPs having main effects only and two SNPs having interaction effects.

For each scenario, 100 replicate datasets were generated with 500 cases and 500 controls and MAF = 0.1, 0.2, 0.3, or 0.4 at all SNPs. Genotypes were generated assuming independence among SNPs and Hardy-Weinberg equilibrium. Quantitative phenotypes were generated conditional on genotypes under a linear model, to reflect an underlying quantitative trait, and affection status was assigned using a threshold. Phenotype data were thus generated under the following probit model:

(5) $$\begin{array}{l} Y=\beta_{0}+\beta_{1}X_{1}+\beta_{2}X_{2}+\beta_{3}X_{3}+\beta_{4}X_{4}+\beta_{5}X_{3}X_{4}+E\\ D=I\left(Y>m\right) \end{array}$$

where E ~ N(0,σ^2^), β_0_ = 20, σ^2^ = 10, and the threshold *m* was chosen to be median(*Y*) to achieve P(D) = 0.5 and balanced data. The genotypes at SNP *j* were coded *X*_*j*_ = 0,1,2 reflecting the number of copies of the minor allele, assuming additive allelic effects (on Y). Note that SNPs 1 and 2 have marginal effects only, whereas SNPs 3 and 4 are interacting. We quantified the strength of the simulated marginal and interacting effects in terms of heritability due to a given genetic effect (Equations 1 and 2). Data were generated under five models, where the vector *β* = (*β*_1_, *β*_2_, *β*_3_, *β*_4_, *β*_5_) was chosen to reflect different effect sizes and patterns in terms of total heritability due to the main effect SNPs 1 and 2 ($H_{X1X2}^{2}=H_{M,X1}^{2}+H_{M,X2}^{2}$) and the total heritability due to the interacting SNPs 3 and 4 ($H_{X3X4}^{2}=H_{M,X3}^{2}+H_{M,X4}^{2}+H_{I,X3X4}^{2}$):

Model 1: Similar effects. The total effects of main and interacting SNPs are similar ($H_{X1X2}^{2}\approx H_{X3X4}^{2}$), with *β* = (.9,.9,1.3,1.3,−1.3).

Model 2: Main effects greater. The total effects of SNPs 1 and 2 are greater than effects of SNPs 3 and 4 ($H_{X1X2}^{2}>H_{X3X4}^{2}$), with *β* = (1.0,1.0,1.0,1.0,−1.0).

Model 3: Main effects only. The total heritability is due to SNPs 1 and 2, and SNPs 3 and 4 are not causative ($H_{X3X4}^{2}=0$), with *β* = (.8,.8,0,0,0).

Model 4: Interaction effects greater. The total effects of SNPs 3 and 4 are greater than SNPs 1 and 2 ($H_{X1X2}^{2}<H_{X3X4}^{2}$), with *β* = (.8,.8,1.5,1.5,−1.5).

Model 5: Interaction effects only. The total heritability is due to SNPs 3 and 4, and SNPs 1 and 2 are not causative ($H_{X1X2}^{2}=0$), with *β* = (0,0,1.3,1.3,−1.3).

In Models 1–5, the type of effect is a property of the heritability corresponding to the main effect SNPs 1 and 2, and the interacting SNPs 3 and 4. Models with low heritability (*H*^2^ ≤ 7%) were chosen to reflect realistic effect sizes that could be expected in genetic studies and also to investigate the performance in situations with low power. The specific heritability components depend not only on the *β* parameters, but also on the minor allele frequencies; in particular, the level of marginal heritability of interacting SNPs 3 and 4 varies with MAF (Equation 2) [29]. See ( Table B1 Additional file 2) for the total, marginal, and interaction heritabilities of the simulated datasets.

In Simulation 1, we investigated performance of VIMs and compared variable importance rankings to p-value rankings from logistic regression. VIMs of interest in these simulations were raw MDA, scaled/Liaw MDA, standard deviation of MDA, and Gini importance. “Probability of detection” is reported for each VIM for ‘main’, ‘interacting’, and ‘null’ SNPs, where the probability of detection was estimated by the proportion of times across 100 replicates that each SNP was detected, averaged across all ‘main’, ‘interacting’, and ‘null’ SNPs, respectively.

### Simulation 2: comparing main effect and interaction detection with RF VIMs vs. logistic regression

**Figure 1:**
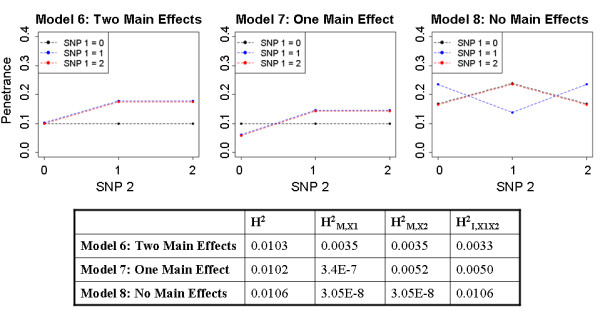
**Simulation 2 penetrance functions.** Penetrance functions for the two locus interactions in the three models used in Simulation 2, with corresponding total, marginal, and interaction heritabilities.

Generally, a threshold or probit model can be approximated by a logit model. This may give logistic regression an advantage in the above Simulation 1 design, as similar models are used for simulating and analyzing the data. In order to provide an additional comparison of RF with univariate logistic regression, data were also generated directly from penetrance functions, the conditional probability of disease given genotypes. For Simulation 2, genotype data were simulated as previously described, with MAF fixed at 0.3 because the effect of MAF had already been examined. Phenotypes were generated conditional on genotypes from a specified penetrance function ( Additional file 2), assuming two true causative loci which interact (SNP 1 and SNP 2). Three model scenarios were considered in which both SNPs contribute marginally (“two main effects” – Model 6), only SNP 2 contributes marginally (“one main effect” – Model 7), and neither SNP contributes marginally to the phenotype (“no main effects” – Model 8). Total genetic effect size was fixed at approximately 1% heritability to facilitate comparison across models. Penetrance functions used for these simulations are provided in Table B2 (Additional file 2) and are displayed in Figure 1, along with the heritability due to each component.

### Simulation 3: investigating detection when LD is present

The goal of the third simulation was to examine the performance of RF VIMs in detecting main and interaction SNP effects under a scenario with realistic patterns of LD and MAF. We therefore generated genotypes based on real data, with various degrees of LD in different regions, and compared performance of interaction detection under some of the previously assumed data-generating models of phenotype conditional on genotype.

A real genome-wide SNP dataset was used as the basis for generating genotypes for *p* = 1,000 SNPs in 56 genes, giving rise to patterns of LD and MAF resembling those encountered in genome-wide association studies. Details of the genetic data simulations are discussed in Additional file 2. Phenotypes from the real genome-wide data were not utilized, and instead binary phenotypes were generated conditional on genotypes under Model 3 (‘main effects only’) and Model 5 (‘interaction effects only’) from Simulation 1 in order to investigate the impact of LD on the performance of RF SNP detection for SNPs with both marginal and interacting effects in the high-dimensional setting (*p* = 1,000). The two causal SNPs were chosen to have MAF of approximately 0.3 to provide results comparable to Simulation 1 without LD. Three patterns of LD were considered, involving causal SNPs either in strong LD (R^2^ > 0.95 with at least three SNPs) or weak LD (R^2^ < 0.3 for all SNPs) with other SNPs; either both causal SNPs were chosen to be in strong LD, both in weak LD, or one in strong and the other in weak LD. A fourth scenario was also considered for comparison where all SNPs were generated independently (i.e. no LD) with MAFs identical to those seen in the real data.

Probability of detection was defined as before, and also as the proportion of times that any SNP in high LD (R^2^ > 0.85) with the causal SNP ranks in the top *k* in order to assess detection of a region around the causal SNP. As in Simulation 1, the performance of RF VIMs was compared to p-value rankings from logistic regression.

## Results and discussion

### Simulation 1: comparing performance of VIMs for detecting main vs. interaction effects

As expected, in general, causal SNPs (both ‘main’ and ‘interacting’) have larger VIMs than null SNPs, particularly for small *p* ( Figure B1, Additional file 2). The causal SNPs also have larger variability in variable importance between trees than null SNPs, as expected.

**Figure 2:**
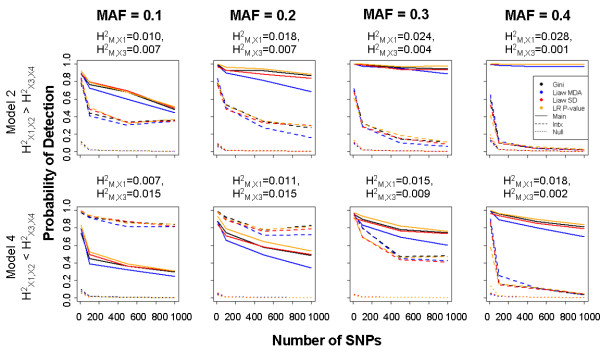
**Simulation 1 results.** Probability of detection for ‘main’, ‘interacting’, and ‘null’ SNPs plotted against the number of total SNPs for select RF VIMs and logistic regression (LR). Top row shows results for the “main effects greater” Model 2; bottom row shows results for “interaction effects greater” Model 4. Results are plotted separately across MAF. Average PE estimates range between 0.430 and 0.476 ( Additional file 2 Table B3).

For all types of SNPs (‘main’, ‘interacting’, and ‘null’), both the estimated variable importance and the probability of detection decline as the total number of predictors increases. However, as the total number of predictors increases, the probability of detection declines more rapidly for interacting SNPs than for non-interacting SNPs (Figure 2; Figures B2-B4, Additional file 2). Only results for Model 2 ($H_{X1X2}^{2}>H_{X3X4}^{2}$) and Model 4 ($H_{X1X2}^{2}<H_{X3X4}^{2}$) are displayed in Figure 2; results for Models 1, 3, and 5 show similar patterns ( Figures B2-B4, Additional file 2). Overall, the detection probability of all SNPs increases with effect size (H^2^), as expected. For example, for the non-interacting SNPs, detection probability increases as H_X1X2_^2^ increases. However, for the interacting SNPs, detection probability is largely dependent upon their marginal effect (marginal heritability, H_M,i_^2^) rather than their total effect (H_X3,X4_^2^), which includes their interaction effect. Thus, detection probability is strongest for the SNPs with the largest marginal heritability (H_M,i_^2^), not necessarily the largest total heritability. For example, under Model 4 with MAF of 0.3, the detection probability is higher for the main effect SNPs than for the interacting SNPs, despite the fact that the total effect of the interacting SNPs (H_X3,X4_^2^) is larger than the effect of the main effect SNPs (H^2^_X1,*X*2_). This is because under this model the main effects SNPs have a larger marginal heritability (0.015) than the interacting SNPs (0.009). Figure 2 demonstrates that the decrease in detection probability for SNPs with small marginal effects becomes more pronounced in situations with large *p*.

A pattern is observed across MAF, where ‘main’ SNPs are more readily detected than ‘interacting’ SNPs for higher frequencies, corresponding to scenarios where H_M,X1_^2^ and H^2^_M,*X*2_ are high. As MAF increases, the difference in detection between ‘main’ and ‘interacting’ SNPs also increases; for low MAFs, ‘interacting’ SNPs are more frequently detected, while for more common variants the ‘main effects’ SNPs are more frequently detected. This is because under our data generating model, as MAF increases the heritability due to the marginal effects of interacting SNPs 3 and 4 (H_M,X3_^2^ and H_M,X4_^2^) decreases ( Table B1, Additional file 2), making them more difficult to detect using RF.

RF prediction errors, under the different simulation models, are shown in Additional file 2 (Table B3). These results demonstrate an inverse relationship between detection probability based on VIMs and the estimates of prediction error for the RF models. While all estimates of prediction error are relatively high (40-50%), which is expected given the low heritability of the assumed models, the estimates of prediction error are lower for models with higher detection probabilities. For models with weak marginal effects (low H_M,i_^2^), the SNP effects are not well detected, and consistent with this, the prediction errors are high (close to 50%).

Of importance is the fact that the probability of detection is not strongly affected by method of ranking (RF VIMs or logistic regression), and in particular the RF VIMs rarely outperform logistic regression for values of *p*>10 for the scenarios studied here (Figure 2; Figures B2-B4, Additional file 2). In all scenarios, logistic regression has slightly higher detection probability for the non-interacting SNPs. When MAF is high (corresponding to situations when the marginal heritability attributable to the ‘interacting’ SNPs is lower than the ‘main’ SNPs; Table B1, Additional file 2), RF variable importance measures have higher probability of detection than logistic regression for interacting SNPs, but only when *p* is low. For instance, Liaw MDA generally has higher detection probability for the ‘interacting SNPs’ when MAF = 0.3, 0.4 if *p* ≤ 100.

### Simulation 2: comparing main effect and interaction detection with RF VIMs vs. logistic regression

**Figure 3:**
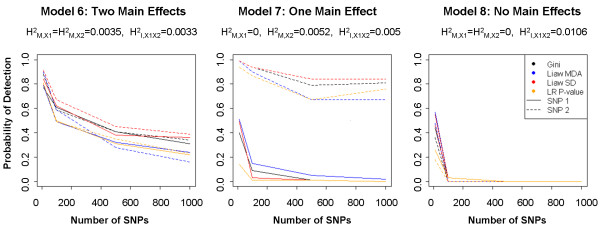
**Simulation 2 results.** Probability of detection for SNP1 and SNP2 plotted against total number of SNPs by VIM for models with interactions and two main effects (Model 6 - left), one main effect (Model 7 - center), and no main effects (Model 8 - right). Average PE estimates range between 0.465 and 0.508 ( Additional file 2 Table B4).

Results for Simulation 2 based on penetrance functions are portrayed in Figure 3, where probability of detection is reported separately for SNPs 1 and 2. When marginal effects are present at both SNPs (Model 6), detection probability is high for both SNPs, but steadily declines as *p* increases. When only SNP 2 exhibits a marginal component (Model 7), detection probability for SNP 2 is high, but detection probability is much lower for interacting SNP 1, particularly for large *p*. When no marginal effects are present at either locus (Model 8), detection probability is near zero when *p*>10. The detection probability is highest when marginal heritability (H_M,i_^2^) is highest, which has a greater impact than method of ranking. No single RF method consistently outperforms the others; however, in general RF VIMs perform slightly better than logistic regression models.

Because total *H*^*2*^ is low for the assumed models, we expect prediction error estimates to be high ( Additional file 2, Table A4). However, the prediction error estimates are particularly high (close to 0.5) for Model 8, which has no marginal effects, indicating that under this model RF cannot detect the SNP effects. Thus, as in Simulation 1, the high prediction error observed under the pure interaction model provides additional evidence that RF was not able to capture the SNP effects in the absence of marginal effects.

### Simulation 3: investigating detection when LD is present

The results of the third set of simulations (Tables 2 and 3) demonstrate how the level of LD impacts the probability of detection for both marginal and interaction effects. For both the two locus model with main effects only (Model 3, Table 2) and with interaction effects (Model 5, Table 3), the detection probability is lower for the causative SNPs in strong LD as compared to the causative SNPs in weak LD for all VIMs. Additionally, strong LD reduces the detection probability of the causal variant far below what can be expected if all genetic predictors are independent, regardless of the type of effect or VIM, although when considering detection of the causal region (rather than the particular SNP) the detection probability is less affected (Tables 2 and 3). For main effect SNPs, the RF VIMs give similar detection probabilities when the causative SNPs are in weak LD with other SNPs and when they are independent (Table 2). However, for interacting SNPs that are in weak LD with other SNPs, the detection probability is higher (Table 3), especially when the other causative SNP is in strong LD with other SNPs (Scenario 3). The Gini importance is more strongly impacted by both strong and weak LD, with greater reductions (with strong LD) and gains (with weak LD) in power than the other importance measures.

**Table 2:** Simulation 3 results, Model 3

	**Level of LD**	**MAF**	**Detection Definition**	**Raw MDA**	**Liaw MDA**	**SD MDA**	**Gini**	**LR P-value**
1	Strong	.294	Causal SNP	0.13	0.12	0.14	0.08	0.21
			Causal Region	0.38	0.3	0.4	0.29	0.49
	Strong	.309	Causal SNP	0.25	0.18	0.28	0.26	0.33
			Causal Region	0.56	0.48	0.55	0.58	0.48
2	Weak	.294	Causal SNP	0.72	0.58	0.73	0.78	0.73
	Weak	.281	Causal SNP	0.66	0.56	0.71	0.79	0.76
3	Strong	.294	Causal SNP	0.15	0.13	0.11	0.08	0.21
			Causal Region	0.5	0.46	0.52	0.39	0.71
	Weak	.294	Causal SNP	0.59	0.52	0.63	0.78	0.5
4	None	.294	Causal SNP	0.67	0.57	0.72	0.73	0.75
	None	.294	Causal SNP	0.68	0.6	0.67	0.7	0.76

Detection probability with and without LD for main effects only Model 3 for RF VIMs and logistic regression (LR). Total number of SNPs = 1,000, MAF ≈ 0.3. Average PE estimates range from 0.458 to 0.477 ( Additional file 2, Table B6).

**Table 3:** Simulation 3 results, Model 5

	**Level of LD**	**MAF**	**Detection Definition**	**Raw MDA**	**Liaw MDA**	**SD MDA**	**Gini**	**LR P-value**
1	Strong	.294	Causal SNP	0.05	0.09	0.05	0.02	0.09
			Causal Region	0.2	0.19	0.21	0.1	0.3
	Strong	.309	Causal SNP	0.2	0.17	0.15	0.16	0.14
			Causal Region	0.47	0.42	0.38	0.41	0.33
2	Weak	.294	Causal SNP	0.43	0.34	0.49	0.52	0.4
	Weak	.281	Causal SNP	0.35	0.25	0.35	0.42	0.29
3	Strong	.294	Causal SNP	0.06	0.09	0.04	0.04	0.12
			Causal Region	0.32	0.27	0.27	0.14	0.41
	Weak	.294	Causal SNP	0.51	0.45	0.4	0.62	0.3
4	None	.294	Causal SNP	0.28	0.21	0.3	0.31	0.33
	None	.294	Causal SNP	0.29	0.27	0.3	0.31	0.29

Detection probability with and without LD for interaction effects only Model 5 for RF VIMs and logistic regression (LR). Total number of SNPs = 1,000, MAF ≈ 0.3. Average PE estimates range from 0.479 to 0.496 ( Additional file 2, Table B6).

In general, the RF VIMs show improved detection over logistic regression for the SNP in weak LD if the other causative SNP is in strong LD (Scenario 3), particularly when the causative genetic factors are interacting (Table 3). In other situations, RF and logistic regression perform similarly when LD is present, with RF performing slightly better under weak LD and logistic regression performing slightly better under strong LD.

### Discussion

In this study, we investigate the ability of Random Forests to detect both marginal and interacting effects in high-dimensional data, in order to validate the claim that RF methods are well suited to describe gene-gene interactions and to determine their usefulness as filter methods or screening tools that allow for interaction effects in large datasets, assuming sample sizes and genetic effect sizes likely to be encountered in real data analysis. While RFs are often cited as an approach suitable for detecting genetic effects in the presence of interactions, McKinney et al. [22] suggested that RFs may be better suited for detecting marginal effects than interactions. However, the effect of data dimensionality on RF’s ability to detect interaction effects has not been previously described. We found that as dimensionality increases, the ability of RF to detect SNP effects diminishes, and this decline is more rapid for interacting SNPs than for SNPs with a large univariate effect. Our results demonstrate that the detection probability of RF is driven by the strength of the marginal components.

In Simulation 1, we observed an inverse relationship between MAF and interaction detection probability, which is a result of the dependency of effect size (i.e. heritability) on MAF. For example, under the data-generating threshold model with stronger interaction effects (Model 4), the marginal effect of the two interacting SNPs (H_M,X3_^2^ and H_M,X4_^2^) decreases and the interaction effect of these two SNPs (H_I,X3X4_^2^) increases, as the MAF increases ( Table B1, Additional file 2). In the case of MAF = 0.1 and 0.2, the marginal effects of the interacting SNPs are strong, and the RF VIMs perform well. However, as MAF increases and these marginal effects diminish, RF has a low probability of detecting the interacting SNPs for large *p*. This demonstrates that in high-dimensional settings RF VIMs are driven by the magnitude of the marginal effects (H_M_^2^), regardless of the presence of an interaction effect (H_I_^2^), and that VIMs are capturing marginal effects rather than interactions as originally claimed. This was also clearly demonstrated by Simulation 2, with models generated from penetrance functions with MAF fixed at 0.3. SNPs that had some level of marginal heritability had higher detection probability, whereas interacting SNPs with no marginal contribution to the total heritability were rarely detected, if at all.

In our simulations, we also observed a strong inverse relationship between estimated prediction error and detection probability. This relationship is expected, since if the RF model was not predictive of phenotype, then no predictive signal was detected for any variable, and hence the true causative factors (if they exist) were not identified. Therefore we do not advocate utilizing a ranked list to screen predictors if prediction error is high, because even if true causative factors exist, they will not be highly ranked. Nevertheless, the relationship between prediction error and detection probability based on VIMs portrays a consistent story: prediction error estimates are only lower than what is expected by chance if the true causal effects are detected. As dimension becomes large, detection probability diminishes and becomes highly dependent on the strength of the marginal effects, and the poor prediction errors are a reflection on the failure of RF to model interactions in these scenarios.

The models used for simulating the data had low *H*^*2*^ to reflect realistic effect sizes for a study of common SNP variants assessed with a genome-wide platform. It has been shown that SNPs identified thus far through GWAS explain only a small portion of the heritability and have poor predictive performance, which is consistent with the models chosen for our simulation study. Nevertheless, we also considered interaction models with stronger effect sizes and higher *H*^*2*^ (data not shown). In these high-heritability models, models with marginal effects resulted in greatly improved prediction error, whereas interaction models without strong marginal effects still showed little if any improvement, reflecting the same general trend described in this study.

The use of alternative definitions of SNP detection and detection probability could impact the findings of this study; however, we found that a previous definition of power utilized by Bureau et al. [20] is similar to our definition in practice and provided similar results. Detection can also be defined using a percentage threshold (i.e. top x% of SNPs) to account for non-constant *p*, but this definition also does not dramatically change the results. Additionally, RF analyses are dependent on various tuning parameters. In our preliminary investigations to select the tuning parameters, we found that increasing the value of *ntree* beyond 5,000 did not improve prediction error or probability of SNP detection. Although increasing the value of *mtry* from 0.1*p* to 0.5*p* resulted in a slight improvement in prediction error, it did not result in a gain in power. Importantly, the observation that as dimensionality increases, the ability of RF to detect interacting SNPs diminishes more rapidly than for SNPs with a large marginal component remained unchanged as *mtry* was increased ( Additional file 1). Hence results of analyses with the value of *mtry* = 0.1*p* are reported here, since this value of *mtry* is more practical for real data analysis as dimension increases, and is the value suggested by Goldstein et al. (2010) [15]. In our application of RF, classification trees were grown by splitting to purity, the current default setting available in software and recommended in the literature. However, some research suggests that this setting may be problematic because the large number of terminal nodes may lead to bias and a loss of consistency in the forest [30,31]. Although the optimal settings of the number of terminal nodes for classification are still unknown, it is possible that a reduced number of terminal nodes could improve results. Further investigation of optimal parameterization of the tree-building algorithm is warranted.

Notably, our simuations revealed that the advantage of RF over univariate logistic regression is lost for larger values of *p*, conflicting with the findings of previous studies. Lunetta et al. (2004) compared the performance of RF variable importance rankings to univariate Fisher’s exact test for *p* = 100 and 1,000 SNPs and found that when interaction effects were present, RF outperformed the univariate method [17]. However, the multiplicative models investigated by Lunetta et al. (2004) have strong marginal components, indicating that the improved performance may be due to the marginal rather than non-linear association. Moreover, the Fisher’s exact test applied by Lunetta et al. is not the most powerful univariate approach, and would rarely be applied in a GWAS setting. They also reported that for the purely epistatic datasets of Ritchie et al. (2003), interaction effects were identified with RF, but these scenarios were low-dimensional with *p* = 10 [32]. In fact, in a methods comparison between multifactor dimensionality reduction, neural networks, and RF for purely epistatic models with *p* = 100 total loci, RF was shown to have extremely low power [33]. In the current study, a broader range of disease models with varying effect types and dimensionality were considered to fully explore performance of RF as dimension increases. Under the Simulation 1 threshold model, we failed to see an improvement with RF over logistic regression for any VIMs. In Simulation 2, the most commonly used VIM (Liaw MDA) again displayed little advantage over p-values from logistic regression, particularly for higher *p*. In contrast, the less commonly used Gini importance and Liaw SD showed a slight advantage over logistic regression in detecting marginal effects.

The results of the study indicate that as a tool for variable selection, both RF VIMs and univariate logistic regression can detect SNPs with marginal components, but neither may be adequate for interaction detection in high dimensions. In lower dimensions, RFs capture interactive effects and may therefore outperform univariate logistic regression. However, in lower dimensions higher order logistic regression models and pair-wise scans are possible, limiting the advantage of RF. In fact, some researchers feel that interactions modeled by RF but not confirmed with logistic regression are unlikely to be real. Nevertheless, the advantages of RF reside in the ability to incorporate the effects of multiple variables simultaneously and model conditional associations in both low and high dimensional data (even if interactions may not be specifically modeled), which cannot be captured with univariate procedures. Thus RF is recommended as a complimentary approach to other variable selection methods. Moreover, we note that machine learning methods such as RF were designed to improve prediction rather than variable selection; therefore if the research objective is to develop a predictive model, then RF may be more appropriate.

Bureau et al. [20] previously recognized that RF VIMs do not explicitly test for interactions, and proposed joint importance measures based on permuting pairs of SNPs to identify pair-wise interactions. However, this joint permutation is computationally intractable in high-dimensional data, even for pair-wise interactions. The hope is that in genome-wide data, the original VIMs may still capture complex associations. Yet we found that RFs fail to capture interactions in high-dimensional settings. While RF seems to identify interactions in low-dimensional data, why do these properties fail to extend to higher dimensions? Unlike univariate analyses, RFs take additional factors into account and model the conditional relationships between variables within a tree. The decision trees in RF can efficiently detect conditional dependencies between predictors that are in the same tree, and therefore interactions are modeled in low-dimensional settings when most variables tend to be present in each tree. However, there is a limit to the depth of a tree based on sample size, and therefore in high-dimensional settings not every variable will make it into a given tree—variables with strong marginal associations are more likely to be chosen for a given split. The probability that a pair of interacting SNPs are in the same tree together is low, particularly if the marginal effects of both SNPs are relatively small; if a pair of SNPs are rarely present in the same tree, then their conditional association is rarely modeled. Research on the asymptotic theory of RF has shown that for a fixed number of predictors *p*, RF is sparse (i.e. depends only on the true number of causal predictors, not the total *p*) as the sample size approaches infinity [34]. However, our results demonstrate that with practical sample size limitations, RF has little ability to detect interactions.

Increasing the sample size tends to increase the tree-depth and number of possible splits per tree, which increases the number of variables included per tree and the probability that the effects of a pair of interacting SNPs will be jointly modeled. To investigate the impact of sample size on power, we considered a difficult genetic model with low power (Model 8) and increased the sample size from *N* = 1,000 to *N* = 5,000 and 10,000. We found that increasing the sample size did increase power (as expected), particularly for *p* = 10 and 100; however, even with *N* = 10,000 power is still extremely low when *p* = 1,000, indicating that to detect an interaction with low heritability and no marginal effects, sample sizes much greater than 10 times the number of predictors are necessary (for full results see Additional file 2). In practice, sample size is often *N < p*, hindering the ability of interaction detection of any statistical method, including RF.

Poor SNP effect detection in high-dimensional data is exacerbated in the presence of strong LD, both for marginal and interaction effects. If the true causative SNPs are in regions of strong LD, the causative effects must compete with correlated predictors for positions in each tree, since non-causative variables may also be associated with the phenotype because of LD. The result is lower importance rankings and a reduction in the probability of SNP detection. Our results were similar to those observed in previous studies, which also found that the presence of SNPs that are highly correlated with risk SNPs reduces RF performance [27,35]. Although SNP detection is reduced for predictors in strong LD, predictors in weak LD show improved results, particularly for interacting SNPs. In fact, the strong LD of one causative factor can improve the detection probability of other causative factors, including interactions. If one risk SNP is highly correlated with other predictors, this increases the probability that additional risk SNPs will appear in a tree together with at least one SNP in the first causative region, increasing the chance of modeling a joint association. This may have implications for LD pruning with RFs, which may be helpful for detecting marginal effects but in certain situations could reduce the chance of detecting effects of interacting SNPs. These observations raise questions about the most appropriate use of RF for genetic datasets.

The results of this study allow us to draw a number of conclusions about the performance of RF, but some inherent limitations remain. In the current study we only considered simple disease models with architectures involving marginal effects and two-locus interactions, and investigation of more complex architectures is warranted. Nevertheless, our results demonstrate difficulties with detecting even these simple lower-order interactions using RF. Furthermore, only a limited number of LD patterns were investigated. Although this was not a thorough examination of the consequences of LD on interaction detection, our results provide some insight into the impact of LD on the performance of RF for identifying interacting SNPs. While beyond the scope of the current study, these findings motivate further research into how RF should be applied in practice for different types of data.

These results call into question the applicability of RF as a variable selection and screening tool in a GWAS setting. In high-dimensional data, true causal SNPs without a strong marginal component are not highly ranked by the variable importance measures, indicating little potential improvement of RF as a filter approach over current univariate techniques. Therefore, extensions that improve the detection of interacting factors would be highly advantageous. As the RF methodology currently stands, the primary goal is not identification of interactions. Because the method incorporates conditional effects, allows for the analysis of high-dimensional data where the number of predictors far exceeds the sample size, and provides a ranking scheme to implement potential filtering, it seems that extension to better capture interaction effects seems promising. This work provides insight into why RF variable importance measures fail to capture interactions in a high-dimensional setting, which motivates further research to develop new variable importance measures to properly account for interacting variables or to modify the approach for accurate variable selection in the presence of interactions.

## Conclusions

The ability of Random Forests variable importance measures to detect interaction effects has not been previously investigated in high-dimensional data. We found that as dimensionality increases, the probability of detection declines more rapidly for interacting SNPs than for non-interacting SNPs and Random Forests no longer outperforms univariate logistic regression. Random Forests efficiently model complex relationships including interactions in low dimensional data, but in high dimensional data they only effectively identify genetic effects with a marginal component. Therefore current variable importance measures may not be useful as filter techniques to capture nonlinear effects in genome-wide data and extensions are necessary to better characterize interactions.

## Competing interests

The authors declare that they have no competing interests.

## Authors’ contributions

SJW designed the study, performed the simulations and data analysis, and drafted the manuscript. CC executed the simulations and facilitated data analysis. RRF, XW, MDA and MH participated in the design of the study and interpretation/presentation of results. JMB conceived of the study, assisted in its design, and drafted the manuscript. All authors read and approved the final manuscript.

## Supplementary Material

Additional file 1 **Supplementary information, preliminary simulations to select tuning parameters.** Descriptions and results of parameter sweeps to determine optimal values of the tuning parameters *mtry* and *ntree* to minimize prediction error and maximize power [13,15]. Click here for file

Additional file 2 **Supplementary information, simulations 1–3.** Additional descriptions and results for Simulations 1–3 [36-38]. Click here for file
