# Supplementary material for: SNP interaction detection with Random Forests in high-dimensional genetic data
Source: BMC Bioinformatics. 2012 Jul 15;13:164. doi: 10.1186/1471-2105-13-164 (PMC3463421; doi:10.1186/1471-2105-13-164)
Supplement: Additional file 1 — Supplementary information, preliminary simulations to select tuning parameters. Descriptions and results of parameter sweeps to determine optimal values of the tuning parameters mtry and ntree to minimize prediction error and maximize power [13,15]. [file 1471-2105-13-164-S1.doc]

# Preliminary Simulations to Select Tuning Parameters

### Methods

The objective of the preliminary simulations was to determine which values of the tuning parameters *mtry* and *ntree* yielded optimal performance and should therefore be used in subsequent analyses. We ran parameter sweeps on the models described in Simulations 1-3 to assess tuning parameter selection; values of *ntree*=1000, 5000, 10000 and *mtry* = √*p*, 2√*p*, 0.1*p*, 0.5*p*, *p* were considered. For each scenario, 100 replicate datasets were generated with 500 cases and 500 controls and *p*=10, 100, 500, or 1000 SNPs.

For each value of mtry and ntree, RF was fit and the prediction error (PE) and probability of detection were calculated. Parameters were selected to minimize PE (as would be done in practice) and to maximize power of detection (our response variable of interest for this study).

### Results and Discussion

*Ntree*=1000 led to slightly higher prediction error than *ntree*=5000, but no reduction in PE (or increase in power) was observed by increasing *ntree* to 10000 (Figure A1). *Therefore* ntree=5000 was chosen for all analyses. Results were similar across all models and are displayed in Figure A1 for Model 1 only for simplicity. Although *ntree*=1000 may be enough to minimize prediction error, more trees are needed to achieve stable estimates of variable importance. Thus *ntree*=5000 was chosen because it had higher power to detect the causal SNPs (data not shown).

For *p*>10, a slight reduction in PE was observed as the value of *mtry* increased, with a plateau and even slight increase after *mtry*=0.5*p* (Figure A1); results are displayed for Model 1 with MAF=0.1 and 0.4, but similar patterns were observed for the other models. For *p*=10, higher values of *mtry* result in highly correlated trees which diminishes performance of RF. Amalgamating multiple uncorrelated trees is a key motivation behind RF and an advantage of RF that results in many beneficial mathematical and statistical properties. Therefore it is important to avoid very high values of *mtry* as this raises tree correlation . We found that a value of *mtry*=0.5*p* is optimal in terms of prediction error in most settings (although as dimension increases beyond *p*=1000, this value becomes more and more computationally intractable).

However, we also observe that in terms of probability of detection (our outcome of interest), there appears to be little, if any, advantage in increasing *mtry* from 0.1*p* to 0.5*p* (Figure A2; results are displayed for Model 1, but patterns are similar across other models). For *p*>10, little improvement in power is observed by increasing *mtry* beyond 0.1*p*; for *p*=10, differences are only observed for the Gini index, due to the large type 1 error for large values of *mtry*. Because the focus of this study is on the relationship between probability of detection and high data dimensionality, and because a value of 0.5*p* is computationally inefficient for high-dimensional GWAS data, we select an optimal value of *mtry*=0.1*p*. Additionally, this value was recommended for analysis of genome-wide data with RF by Goldstein et al and reflects a value representative of what would usually be used in real data analysis of high-dimensional data. Hence the optimal tuning parameter settings were determined to be *mtry*=0.1*p* and *ntree*=5000 for subsequent simulation studies.

We also note that a value of *mtry*=0.1*p* vs. 0.5*p* does not change our overall results (Figure A3; results are shown for Model 1 only, but other figures are similar). The main conclusions seen in Simulations 1 and 2—that the difference in detection probability between ‘main’ and ‘interacting’ SNPs increases with *p* and the probability of detection declines more rapidly for ‘interacting’ SNPs than for marginal SNPs—are still observed for either value of *mtry* (0.1*p* or 0.5*p*) (see Figure A3).


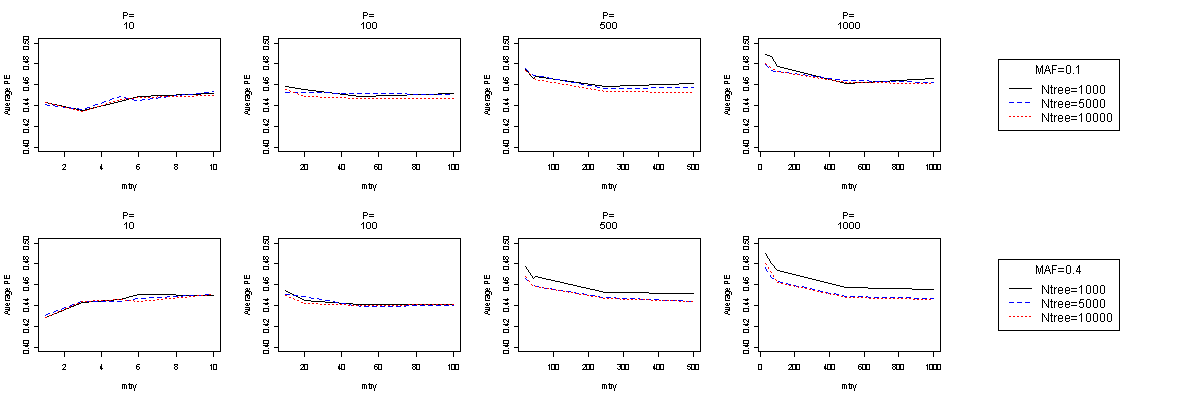


**Figure A1:** Average Prediction Error for Model 1 by *ntree* (plotted against *mtry*), for MAF=0.1 and 0.4.


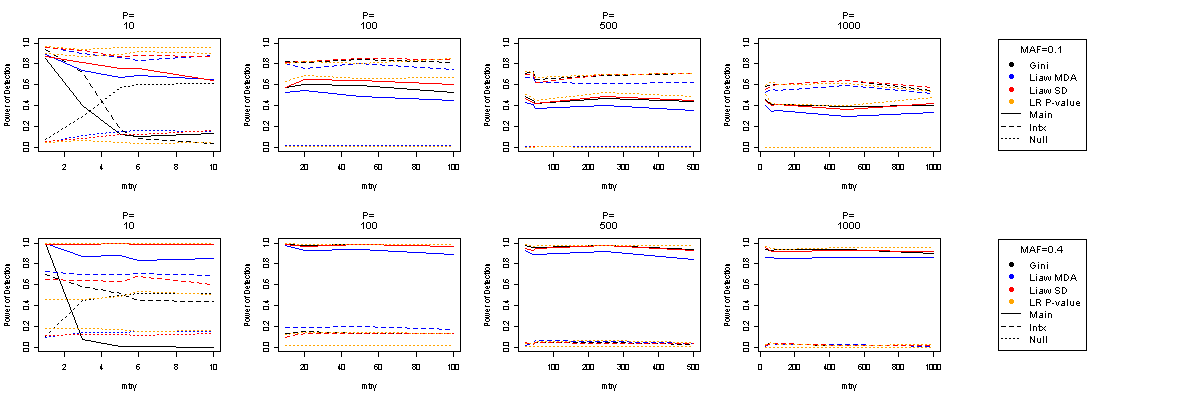


**Figure A2:** Probability of detection for Model 1 by *mtry* for *ntree*=5000, MAF=0.1 and 0.4.


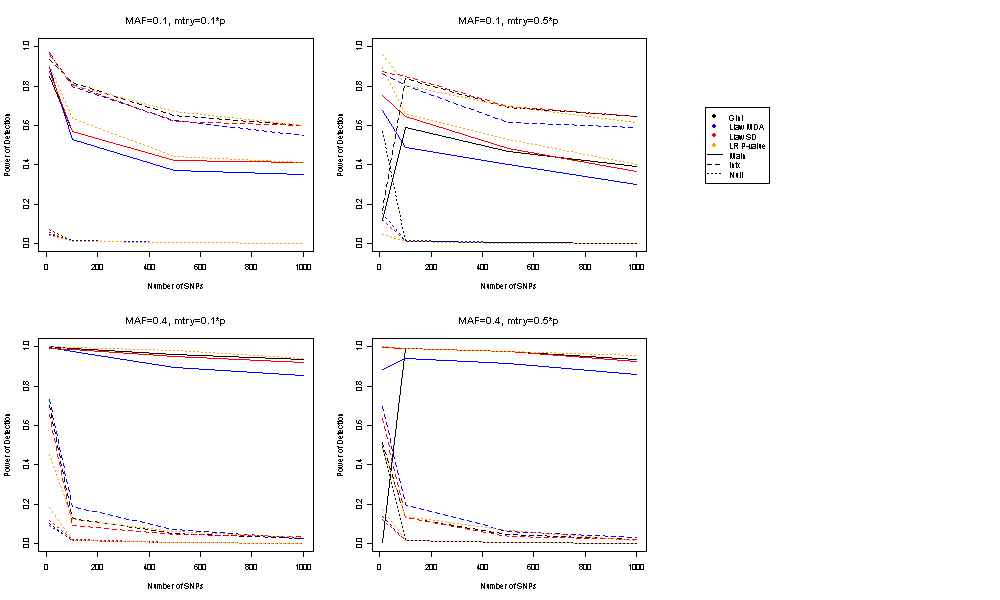


**Figure A3:** Probability of Detection for Model 1 by *p* for *mtry*=0.1*p* vs 0.5*p*. MAF=0.1 top row, MAF=0.4 bottom row.

# References

13. Breiman L: **Random Forests**. *Mach Learn* 2001, **45**:5-32.

15. Goldstein BA, Hubbard AE, Cutler A, Barcellos LF: **An application of Random Forests to a genome-wide association dataset: methodological considerations & new findings**. *BMC genetics* 2010, **11**:49.
