# Supplementary material for: SNP interaction detection with Random Forests in high-dimensional genetic data
Source: BMC Bioinformatics. 2012 Jul 15;13:164. doi: 10.1186/1471-2105-13-164 (PMC3463421; doi:10.1186/1471-2105-13-164)
Supplement: Additional file 2 — Supplementary information, simulations 1–3. Additional descriptions and results for Simulations 1–3 [36-38]. [file 1471-2105-13-164-S2.doc]

# Supplementary Information, Simulations 1-3

# Methods

## Simulation 1 Design

**Table B1:** Total, marginal, and interaction heritabilities* for Simulation 1 Models 1-5 by minor allele frequency.

|  | **MAF** | **H^2^** | **H^2^_M,X1_** | **H^2^_M,X2_** | **H^2^_M,X3_** | **H^2^_M,X4_** | **H^2^_I,X3X4_** | **H^2^_X3X4_** | **H^2^_X1X2_** |
| --- | --- | --- | --- | --- | --- | --- | --- | --- | --- |
| **Model 1** | 0.1 | 0.043 | 0.008 | 0.008 | 0.011 | 0.011 | 0.003 | 0.026 | 0.017 |
|  | 0.2 | 0.062 | 0.015 | 0.015 | 0.011 | 0.011 | 0.010 | 0.033 | 0.029 |
|  | 0.3 | 0.069 | 0.019 | 0.019 | 0.006 | 0.006 | 0.017 | 0.030 | 0.039 |
|  | 0.4 | 0.070 | 0.022 | 0.022 | 0.002 | 0.002 | 0.022 | 0.026 | 0.045 |
| **Model 2** | 0.1 | 0.036 | 0.010 | 0.010 | 0.007 | 0.007 | 0.002 | 0.016 | 0.021 |
|  | 0.2 | 0.055 | 0.018 | 0.018 | 0.007 | 0.007 | 0.006 | 0.019 | 0.036 |
|  | 0.3 | 0.066 | 0.024 | 0.024 | 0.004 | 0.004 | 0.010 | 0.018 | 0.048 |
|  | 0.4 | 0.070 | 0.028 | 0.028 | 0.001 | 0.001 | 0.013 | 0.015 | 0.055 |
| **Model 3** | 0.1 | 0.014 | 0.007 | 0.007 | 0.000 | 0.000 | 0.000 | 0.000 | 0.014 |
|  | 0.2 | 0.025 | 0.013 | 0.013 | 0.000 | 0.000 | 0.000 | 0.000 | 0.025 |
|  | 0.3 | 0.033 | 0.016 | 0.016 | 0.000 | 0.000 | 0.000 | 0.000 | 0.033 |
|  | 0.4 | 0.036 | 0.018 | 0.018 | 0.000 | 0.000 | 0.000 | 0.000 | 0.036 |
| **Model 4** | 0.1 | 0.047 | 0.007 | 0.007 | 0.015 | 0.015 | 0.004 | 0.034 | 0.013 |
|  | 0.2 | 0.066 | 0.011 | 0.011 | 0.015 | 0.015 | 0.013 | 0.043 | 0.023 |
|  | 0.3 | 0.070 | 0.015 | 0.015 | 0.009 | 0.009 | 0.022 | 0.040 | 0.031 |
|  | 0.4 | 0.069 | 0.018 | 0.018 | 0.002 | 0.002 | 0.029 | 0.034 | 0.035 |
| **Model 5** | 0.1 | 0.026 | 0.000 | 0.000 | 0.012 | 0.012 | 0.003 | 0.026 | 0.000 |
|  | 0.2 | 0.033 | 0.000 | 0.000 | 0.011 | 0.011 | 0.010 | 0.033 | 0.000 |
|  | 0.3 | 0.030 | 0.000 | 0.000 | 0.006 | 0.006 | 0.017 | 0.030 | 0.000 |
|  | 0.4 | 0.024 | 0.000 | 0.000 | 0.002 | 0.002 | 0.021 | 0.024 | 0.000 |

* Heritabilities are calculated based on Equations 1-3 and depend on the disease penetrance values P(D|G) and genotype frequencies P(G).

Genotype frequencies are calculated based on the assumed MAF and the assumption of HWE. Disease penetrance values for each of the 81 genotype combinations for the 4 causal loci can be calculated from the specified probit model in Equation 3, where $P(D=1|X)=P(Y>m|X)=\Phi\left(-\left(\frac{y^{*}-m}{\sigma}\right)\right)$ where y*=Xβ, with X=(1,X_1_,X_2_,X_3_,X_4_, X_3_*X_4_) and β=(β_0_, β_1_, β_2_, β_3_, β_4_, β_5_). The median *m* depends on the distribution of Y, which is controlled by β, σ, and MAF.

## Simulation 2 Design

**Table B2:** Penetrance Functions for Simulation 2.

A: Model 6, Two main effects*

|  | SNP2=0 | SNP2=1 | SNP2=2 |
| --- | --- | --- | --- |
| SNP1=0 | α = 0.1 | α = 0.1 | α = 0.1 |
| SNP1=1 | α = 0.1 | α+β = 0.175 | α+β = 0.175 |
| SNP1=2 | α = 0.1 | α+β = 0.175 | α+β = 0.175 |

*α and β were chosen such that H^2^≈0.01, H^2^_M,X1_≈H^2^_M,X2_≈H^2^_I,X1X2_≈ H^2^/3

B: Model 7, One main effect*

|  | SNP2=0 | SNP2=1 | SNP2=2 |
| --- | --- | --- | --- |
| SNP1=0 | α = 0.1 | α = 0.1 | α = 0.1 |
| SNP1=1 | α-γ = 0.057 | α+β = 0.142 | α+β = 0.142 |
| SNP1=2 | α-γ = 0.057 | α+β = 0.142 | α+β = 0.142 |

*α, β, and γ were chosen such that H^2^≈0.01, H^2^_M,X1_=0, H^2^_M,X2_≈H^2^_I,X1X2_≈ H^2^/2

C: Model 8, No main effects*

|  | SNP2=0 | SNP2=1 | SNP2=2 |
| --- | --- | --- | --- |
| SNP1=0 | α = 0.165 | α+β = 0.235 | α = 0.165 |
| SNP1=1 | α+β = 0.235 | α-γ = 0.138 | α+β = 0.235 |
| SNP1=2 | α = 0.165 | α+β = 0.235 | α = 0.165 |

*α, β, and γ were chosen such that H^2^=H^2^_I,X1X2_≈0.01, H^2^_M,X1_=H^2^_M,X2_=0

## Simulation 3 Data Generation

Genotypes were generated using the R package HapSim , which requires input of genetic data representing an underlying population. As the reference population data, we used a subset of genome-wide SNP data from the Study of Addiction: Genetics and Environment (SAGE) , which are available through dbGaP. This dataset was used only as a source of genotypes with realistic LD patterns to generate genotype data, and the phenotype information was not used; instead, causal loci were selected and phenotypes were generated under Models 3 and 5 based on the genotypes at these loci. To create a dataset with 1000 SNPs, we selected SNPs from one gene pathway. In particular, we selected genes that belong to the basal carcinoma pathway, which consists of 1028 SNPs in 56 genes, with average gene size of 18.3 SNPs. The MAF of the SNPs ranged from 0.010 to 0.499. The first 1000 SNPs in the pathway (in chromosomal order) were used as the basis for simulating genetic data. The haplotype distribution was estimated using fastPHASE , and pairs of haplotypes were sampled using HapSim, generating genotypes with patterns of minor allele frequency and linkage disequilibrium resembling the original data.

# Results

## Additional Results, Simulation 1

**Table B3:** Average Prediction Error estimates for Simulation 1 Models 1-5 by model type, MAF, and number of SNPs; *mtry*=0.1*p*, *ntree*=5000.

| **Model** | **MAF** | **P=10** | **P=100** | **P=500** | **P=1000** |
| --- | --- | --- | --- | --- | --- |
| Model 1 | 0.1 | 0.441 | 0.452 | 0.469 | 0.473 |
|  | 0.2 | 0.426 | 0.442 | 0.458 | 0.462 |
|  | 0.3 | 0.425 | 0.444 | 0.460 | 0.459 |
|  | 0.4 | 0.430 | 0.451 | 0.458 | 0.463 |
| Model 2 | 0.1 | 0.448 | 0.467 | 0.476 | 0.477 |
|  | 0.2 | 0.429 | 0.447 | 0.453 | 0.463 |
|  | 0.3 | 0.424 | 0.441 | 0.456 | 0.463 |
|  | 0.4 | 0.423 | 0.447 | 0.448 | 0.448 |
| Model 3 | 0.1 | 0.478 | 0.495 | 0.496 | 0.497 |
|  | 0.2 | 0.461 | 0.476 | 0.476 | 0.485 |
|  | 0.3 | 0.452 | 0.462 | 0.474 | 0.479 |
|  | 0.4 | 0.445 | 0.458 | 0.473 | 0.477 |
| Model 4 | 0.1 | 0.435 | 0.444 | 0.460 | 0.464 |
|  | 0.2 | 0.429 | 0.447 | 0.455 | 0.461 |
|  | 0.3 | 0.427 | 0.451 | 0.464 | 0.470 |
|  | 0.4 | 0.437 | 0.457 | 0.467 | 0.476 |
| Model 5 | 0.1 | 0.450 | 0.472 | 0.484 | 0.483 |
|  | 0.2 | 0.446 | 0.472 | 0.477 | 0.487 |
|  | 0.3 | 0.464 | 0.482 | 0.496 | 0.501 |
|  | 0.4 | 0.488 | 0.498 | 0.501 | 0.506 |


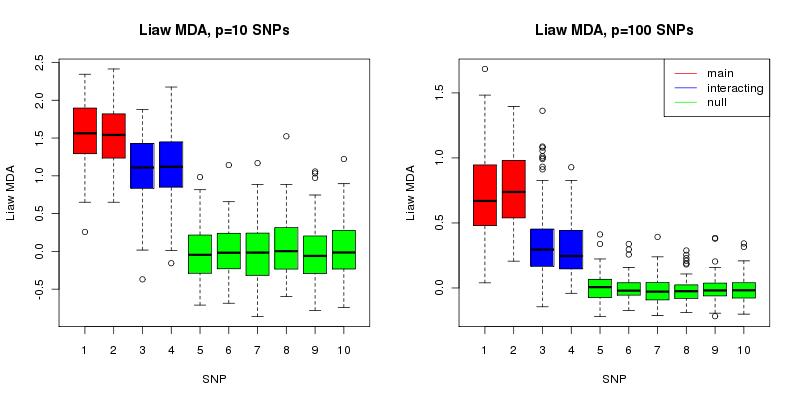


**Figure B1:** Boxplots of average Liaw importance (the most commonly used RF VIM) for SNPs 1-10 for *p*=10 (left) and *p*=100 (right), for Model 1 with MAF=.3. Other VIMs display similar results.


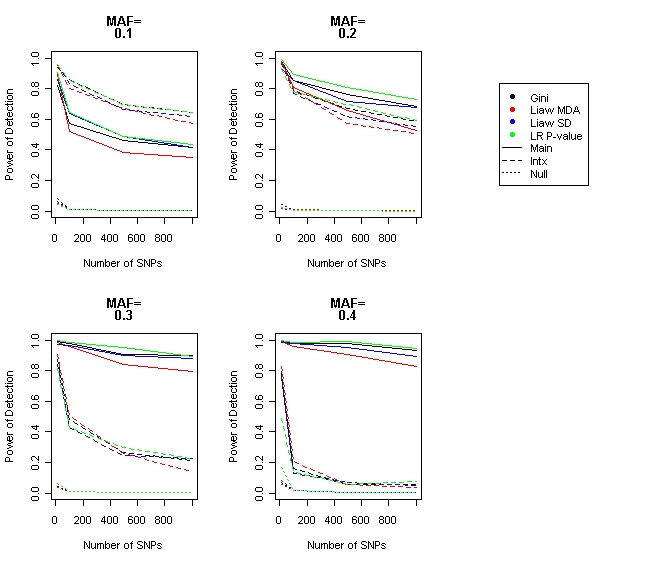


**Figure B2:** Probability of Detection for Simulation 1, Model 1 (Similar effect sizes). Probabilities are plotted against *p* for “main”, “interacting”, and “null” SNPs by VIM.


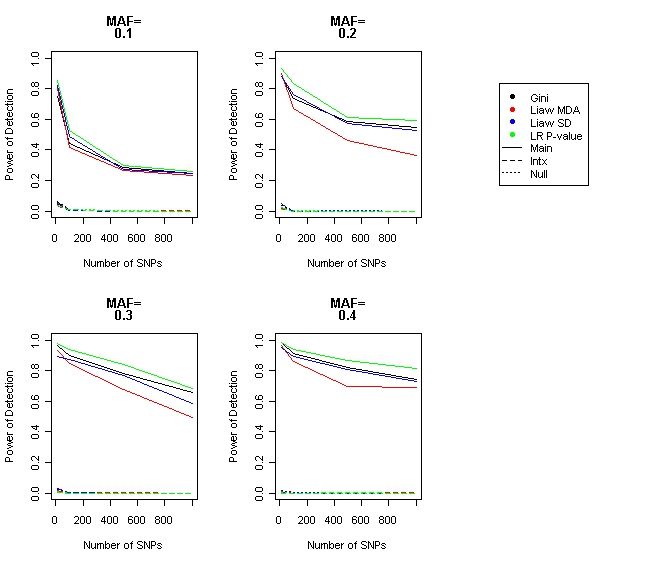


**Figure B3:** Probability of Detection for Simulation 1, Model 3 (Main effects only).

Probabilities are plotted against *p* for “main”, “interacting”, and “null” SNPs by VIM.


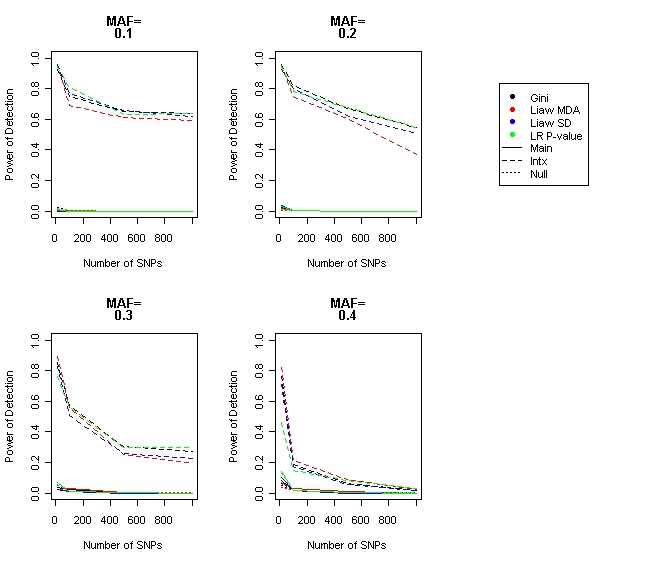


**Figure B4:** Probability of Detection for Simulation 1 Model 5 (Interaction effects only).

Probabilities are plotted against *p* for “main”, “interacting”, and “null” SNPs by VIM.

## Additional Results, Simulation 2

**Table B4:** Average prediction error estimates for Models 6-8 by number of SNPs; total model heritability=1% and *N*=1000.

| **Model** | **P=10** | **P=100** | **P=500** | **P=1000** |
| --- | --- | --- | --- | --- |
| Model 6: Two main effects | 0.465 | 0.482 | 0.492 | 0.493 |
| Model 7: One main effect | 0.466 | 0.479 | 0.488 | 0.490 |
| Model 8: No main effects | 0.496 | 0.501 | 0.508 | 0.507 |

**Table B5:** Average prediction error results for Model 8 by total number of SNPs and sample size *N*.

| **Sample Size** | **P=10** | **P=100** | **P=500** | **P=1000** |
| --- | --- | --- | --- | --- |
| N=1000 | 0.496 | 0.501 | 0.508 | 0.507 |
| N=5000 | 0.487 | 0.498 | 0.502 | 0.501 |
| N=10000 | 0.48 | 0.499 | 0.501 | 0.502 |


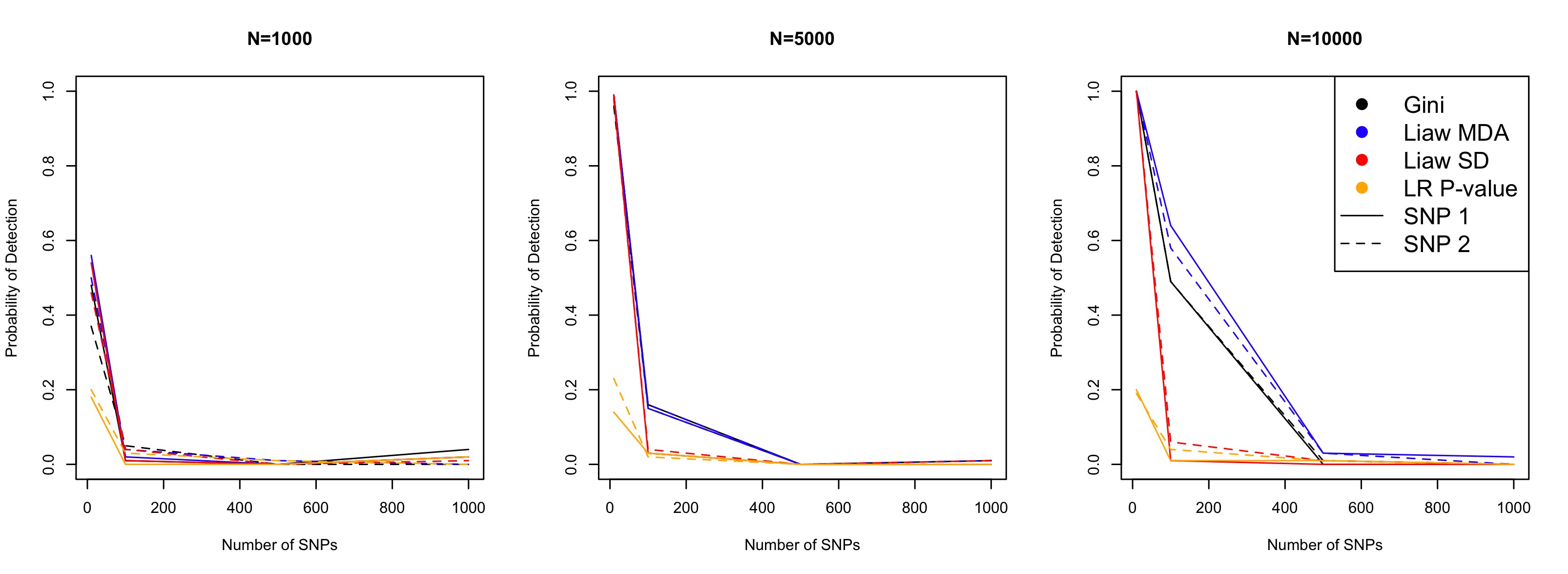


**Figure B5:** Power results, large *N* scenarios (No main effects, Model 8). Probability of detection for SNP1 and SNP2 plotted against total number of SNPs by VIM for Simulation 2 Model 8 (No Main Effects) for *N*=1000 (left), 5000 (center) and 10000 (right).

## Additional Results, Simulation 3

**Table B6:** Average prediction error results for Simulation 3 Models 3 and 5 by pattern of LD of the two causal SNPs.

| **LD Pattern of Causal SNPs** | **Model 3** | **Model 5** |
| --- | --- | --- |
| **2 Strong** | 0.458 | 0.479 |
| **2 Weak** | 0.477 | 0.494 |
| **1 Strong, 1 Weak** | 0.465 | 0.485 |
| **Independent** | 0.475 | 0.496 |

# References

36. Montana G: **HapSim: a simulation tool for generating haplotype data with pre-specified allele frequencies and LD coefficients**. *Bioinformatics* 2005, **21**(23):4309-4311.

37. Bierut LJ, Agrawal A, Bucholz KK, Doheny KF, Laurie C, Pugh E, Fisher S, Fox L, Howells W, Bertelsen S *et al*: **A genome-wide association study of alcohol dependence**. *Proceedings of the National Academy of Sciences of the United States of America*, **107**(11):5082-5087.

38. Scheet P, Stephens M: **A fast and flexible statistical model for large-scale population genotype data: Applications to inferring missing genotypes and haplotypic phase**. *Am J Hum Genet* 2006, **78**(4):629-644.
